# Age-Dependent Changes in the Inflammatory Nociceptive Behavior of Mice

**DOI:** 10.3390/ijms161126041

**Published:** 2015-11-18

**Authors:** Tanya S. King-Himmelreich, Christine V. Möser, Miriam C. Wolters, Katrin Olbrich, Gerd Geisslinger, Ellen Niederberger

**Affiliations:** *pharmazentrum frankfurt*/ZAFES, Institut für Klinische Pharmakologie, Klinikum der Goethe-Universität Frankfurt, Theodor Stern Kai 7, 60590 Frankfurt am Main, Germany; tanya.sarah.king@googlemail.com (T.S.K.-H.); chmoeser@hotmail.com (C.V.M.); wolters@med.uni-frankfurt.de (M.C.W.); k_olbrich@gmx.de (K.O.); geisslinger@em.uni-frankfurt.de (G.G.)

**Keywords:** nociception, inflammation, age, cortisol

## Abstract

The processing of pain undergoes several changes in aging that affect sensory nociceptive fibers and the endogenous neuronal inhibitory systems. So far, it is not completely clear whether age-induced modifications are associated with an increase or decrease in pain perception. In this study, we assessed the impact of age on inflammatory nociception in mice and the role of the hormonal inhibitory systems in this context. We investigated the nociceptive behavior of 12-month-old *versus* 6–8-week-old mice in two behavioral models of inflammatory nociception. Levels of TRP channels, and cortisol as well as cortisol targets, were measured by qPCR, ELISA, and Western blot in the differently aged mice. We observed an age-related reduction in nociceptive behavior during inflammation as well as a higher level of cortisol in the spinal cord of aged mice compared to young mice, while TRP channels were not reduced. Among potential cortisol targets, the NF-κB inhibitor protein alpha (IκBα) was increased, which might contribute to inhibition of NF-κB and a decreased expression and activity of the inducible nitric oxide synthase (iNOS). In conclusion, our results reveal a reduced nociceptive response in aged mice, which might be at least partially mediated by an augmented inflammation-induced increase in the hormonal inhibitory system involving cortisol.

## 1. Introduction

Aging is associated with a number of cellular and neurochemical changes in the nociceptive system that might influence the nociceptive response. It is obvious that chronic pain has a high prevalence in elderly people, but there are also several hypotheses on the potential antinociceptive effects of age. It has been suggested that age-related reduction of peripheral nociceptive fibers and channels might prevent the development of pain hypersensitivity [1], but other studies also showed that age-related declines in neural inhibitory systems such as the opioid system or the cholinergic system might facilitate the occurrence of pain [2,3]. Publications on pain in older individuals reveal contradictory results, showing increased as well as decreased nociceptive responses depending on the models used and the age of the persons/animals [4,5]. In particular, models using reflex-based and operant testing methods deliver partially controversial results; it has been suggested that operant systems might be more consistent with evidence from humans [6]. A switch in the endogenous inhibitory systems might contribute to changes in pain sensitivity and is also controversially discussed. Some studies indicate an age-related decline of endogenous antinociception [7,8], while others suggest an increased non-opioid endogenous pain inhibition in age. The latter has been associated with the hypothalamic–pituitary–adrenal (HPA) axis, e.g., in the tail-flick test in rats (reviewed in [5]). In response to stress, endogenous cortisol levels increase by activation of the HPA axis. Initially, the hypothalamus releases corticotrophin-releasing hormone (CRH), which stimulates the anterior pituitary to release adrenocorticotropic hormone (ACTH). This induces cortisol release in the adrenal cortex. In a negative feedback loop, cortisol downregulates the hormone-releasing activity of both the hypothalamus and the anterior pituitary [9], a mechanism that is impaired in aging [10] and also in the context of inflammation [9,11]. Therefore, the cortisol release is prolonged and might contribute to analgesia in older mice by activation of anti-stress and anti-inflammatory pathways. These effects could be mediated by glucocorticoid-induced inhibition of pro-inflammatory gene expression and an increase of anti-inflammatory mediators, e.g., an increase in the amount of the nuclear factor of kappa light chain enhancer of activated B-cells (NF-κB) inhibitor alpha (IκBα) [12,13], which inhibits NF-κB activity and the immune response.

Since the effects of age on inflammatory nociception are still not clarified, the current study intended to investigate the effect of age in two reflex-based models of inflammatory nociception to provide a better comparison with already published studies in this field. Furthermore, we analyzed cortisol levels and the regulation of the cortisol target proteins IκBα and inducible nitric oxide synthase (iNOS) in the spinal cord of differently aged mice with and without inflammatory noxious stimulation.

## 2. Results

### 2.1. Old Mice Show Reduced Nociceptive Behavior in Inflammatory Models

**Figure 1:**
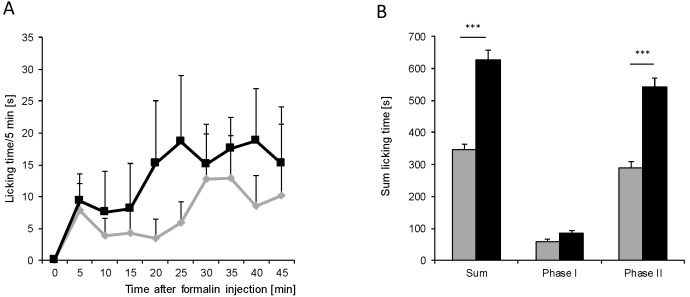
Inflammatory nociception in the Formalin test. (**A**) Time course of the licking behavior in young (■) and old mice (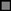) (*n* = 8 mice/group) after injection of formalin (5%, 20 µL) into the hind paw. Formalin was injected at time “0”, and the time spent licking the injected paw was measured at 5 min intervals for 45 min; (**B**) Statistical analysis of total licking time, phase I (0–10 min) and phase II (11–45 min) between young (black column) and old (grey column) mice. *** *p* < 0.001, significant mean difference between young and old mice concerning the total licking time and the licking time in the second phase of the formalin test, respectively.

**Figure 2:**
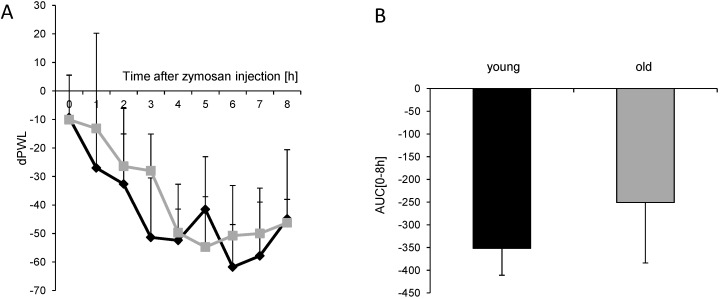
Inflammatory nociception in the zymosan-induced paw inflammation. (**A**) Time course of mechanical hypersensitivity in young (■) and old mice (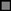) after injection of 10 mg/mL (20 µL) zymosan A into a hind paw. The diagram shows the delta paw withdrawal latencies (ΔPWL) in response to mechanical stimulation, as assessed with a Dynamic Plantar Aesthesiometer; (**B**) Comparison of the area under the paw withdrawal latency *versus* time curve between young (black column) and old (grey column) mice 0 to 8 h after zymosan A injection. (*n* = 6 mice/group).

The nociceptive behavior of old and young mice was compared in two models of inflammatory nociception. In the formalin test as well as zymosan-induced paw inflammation, a reduced nociceptive response was observed in old mice (Figure 1 and Figure 2). In the formalin test, young mice at the age of 6–8 weeks showed the typical biphasic behavior following a 5% formalin injection into the dorsal surface of one hind paw. The first 10 min of nociception are owed to acute pain; the second phase with a maximal pain reaction around 30 min after injection is caused by inflammatory nociception and spinal mechanisms of central sensitization. Older mice at the age of 12 months showed a similar response in the first phase; however, inflammatory nociception in phase 2 was significantly reduced compared to young mice (Figure 1) (age × repeated measures: *F*_(9, 126)_ = 10.36, *p* < 0.0001). After injection of zymosan A into the plantar surface of one hind paw, young mice showed a strong decrease in the paw withdrawal latencies in response to a mechanical stimulus, indicating the development of mechanical hypersensitivity. This response was slightly decreased in the older mice as indicated by age × repeated measures (*F*_(8,72)_ = 8.74, *p* < 0.0001). The area under the curve (AUC) diagram, however, showed no significant differences (Figure 2).

To rule out the decreased sensitivity of old mice being due to motor deficits or decrease of nociceptive fibers and already apparent in acute nociception, we assessed the motor functions in the Hanging wire test and the nociceptive reaction upon acute noxious cold, heat, and mechanical stimulation. In all of these tests, we found no difference between the two groups (Table 1), indicating that motor function and acute nociception are normal in old mice.

**Table 1:** Effects of age on acute nociception.

Test/Age	Old	Young
Hargreaves paw withdrawal latencies (PWL) [s] ± SD	11.5 ± 3.4 (*n* = 8)	10.5 ± 2.8 (*n* = 6)
Dynamic Plantar PWL [s] ± SD	8.2 ± 1.3 (*n* = 8)	8.2 ± 0.6 (*n* = 6)
Hot Plate PWL [s] ± SD	21 ± 2.7 (*n* = 8)	18.2 ± 3.8 (*n* = 4)
Acetone Reaction time/2 min [s] ± SD	2.9 ± 2.7 (*n* = 8)	2.1 ± 1.9 (*n* = 6)

### 2.2. Effect of Age on Central and Peripheral TRP Channels

**Figure 3:**
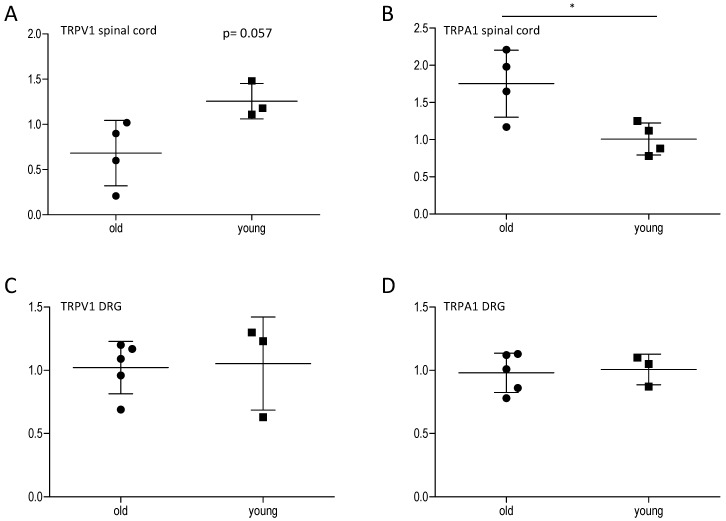
Effect of age on TRPV1 and TRPA1 expression in the spinal cord and the DRGs. TRPV1 and TRPA1 mRNA expression in the spinal cord (**A**,**B**) and the DRGs (**C**,**D**) of young and old mice 45 min after formalin injection into the hind paw, analyzed by qRT-PCR (*n* = 3–5/group), * *p* < 0.05, statistically significant difference between the groups.

Since it has already been suggested that nociceptive sensitivity might decrease in age due to a loss of nociceptive fibers, we investigated the levels of the typical nociceptive genes TRPV1 and TRPA1. These genes are preferentially expressed in sensory nociceptive fibers, which end in the spinal cord, where the nociceptive signal is transduced to the central nervous system. Changes in their expression and activity contribute to peripheral and central sensitization mechanisms [14]. Therefore, we assessed their potential modulation in the spinal cord and in the dorsal root ganglia of young and old mice by qPCR analyses. In the spinal cord, we found a non-significant reduction of TRPV1 and a significant upregulation of TRPA1 in old mice in comparison to young mice. Unexpectedly, in the periphery, there was no difference between old and young mice concerning expression of TRPA1 and TRPV1, indicating that a loss of nociceptive fibers is not necessarily responsible for the decreased nociceptive behavior in old mice (Figure 3).

### 2.3. Effect of Age on Cortisol and Downstream Targets

**Figure 4:**
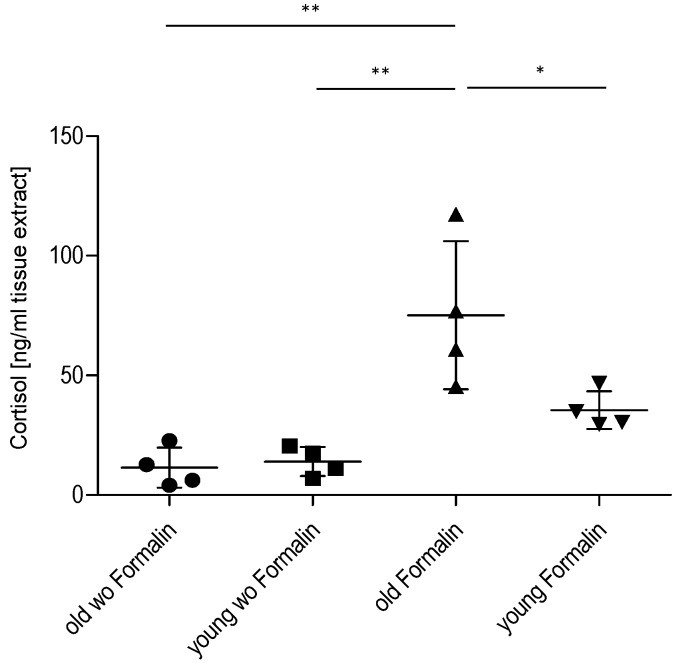
Effect of age on spinal cortisol levels in mice with/without nociceptive stimulation. Cortisol levels in young and old mice at basal level and 45 min after injection of formalin into the hind paw, as assessed by cortisol ELISA (*n* = 4/group). wo = without formalin, * *p* < 0.05, ** *p* < 0.01, statistically significant difference.

**Figure 5:**
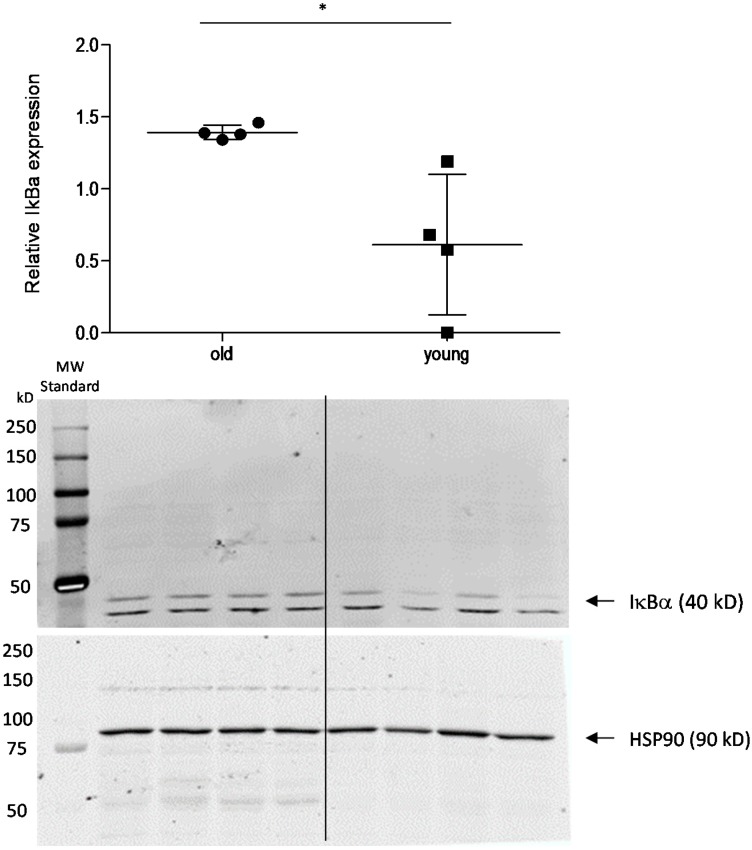
Effect of age on expression of IκBα in mice with nociceptive stimulation. Western blot analysis of IκBα expression in the spinal cord of young and old mice 45 min after injection of formalin into the hind paw. The diagram shows the densitometric analysis of three independent experiments, the blots one representative example, (*n* = 4). Due to scanning the blots at two different wavelengths, the standard is only visible in the IκBα blot. * *p* < 0.05, statistically significant difference between the groups.

**Figure 6:**
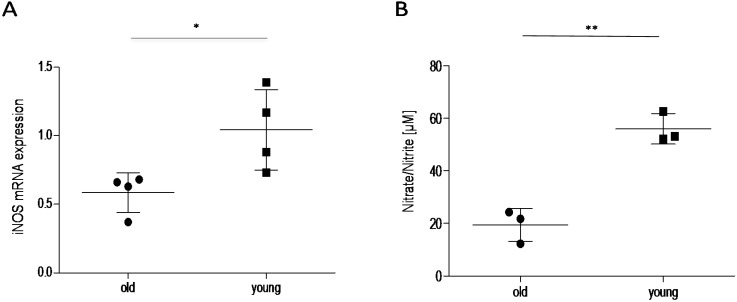
Effect of age on expression and activity of iNOS in the spinal cord of mice 45 min after formalin injection. (**A**) iNOS mRNA expression in the spinal cord of young and old mice 45 min after formalin injection into the hind paw, analyzed by qRT-PCR (*n* = 4/group); (**B**) iNOS activity as assessed by Griess assay, (*n* = 3), * *p* < 0.05, ** *p* < 0.01, statistically significant difference between the groups.

To assess the impact of the HPA axis on the decreased inflammatory nociception in older mice, we assessed spinal cortisol levels using ELISA. Basal control levels of cortisol in the spinal cord were similar in young and old mice. After injection of formalin into the hind paws, the spinal cord cortisol levels increased ~2.5-fold in young and ~7.5-fold in older animals, which is significantly higher in formalin-treated old mice in comparison to control and to young mice (Figure 4). These results indicate that cortisol might contribute to the reduced nociceptive behavior in these mice by modulation of cortisol-dependent, pain-relevant target genes. To address this point, we investigated the cortisol-downstream target IκBα [15,16] by Western blot (Figure 5). As expected, high cortisol levels in older mice are associated with increased IκBα expression, indicating that inhibition of NF-κB activation might contribute to the observed effects. Another well-known cortisol and NF-κB target is the inducible isoform of nitric oxide synthase (iNOS) [17,18]. iNOS expression and activity was analyzed by real-time RT-PCR and determination of the stable NO metabolites nitrate/nitrite (Griess Assay) (Figure 6). In accordance with high cortisol levels and a reduction of the nociceptive response, iNOS expression and activity was reduced in the spinal cord of old mice after the formalin test compared to young mice.

## 3. Discussion

Increasing age is mostly associated with a high prevalence of pain. However, studies investigating this phenomenon often show contradictory results, supporting or opposing augmented pain in age. This study was designed to compare the inflammatory nociceptive response in mice aged 6–8 weeks or 12 months. Our results of the behavioral models showed that older mice exhibit a reduced nociceptive response when compared to young mice. To exclude the possibility that the performance of older mice might be impaired by reduced motor coordination, an increase in body weight, or a proposed loss of sensory nociceptive fibers [1], we examined motor function, acute nociception, and the expression of central and peripheral nociceptive channels. Except for an upregulation of TRPA1 in the spinal cord of old mice, we did not find differences between young and old mice, which indicates that all these functions are normal in old mice and not responsible for the observed reduction in the nociceptive response. Further experiments revealed that the underlying signal transduction mechanisms might at least partially rely on an increase of cortisol production. Cortisol is a glucocorticoid released from the adrenal cortex in response to stress. Its immune-suppressing characteristics are well known and therapeutically utilized. Its potential role in nociception at higher ages is supported by studies showing that the HPA axis with cortisol as effector is associated with stress-induced analgesia [19,20,21,22]. This might be due to a loss of the negative feedback loop of HPA-produced cortisol, which controls the cortisol-releasing activity in younger individuals [10]. Therefore, ongoing higher stress-induced cortisol levels might contribute to the reduced nociceptive behavior of old mice in the inflammatory models, as shown in our study. Basal levels of cortisol in both young and old mice were similarly low. Generally, studies using models of acute, inflammatory, or neuropathic pain show a number of different results including decreased, unaltered, or increased pain sensitivity in aged animals [5,23]. This discrepancy is also reflected in this study. Our results are in line with data that revealed a decrease in mechanical hypersensitivity and a reduced number of spinal microglia in old animals in the zymosan-induced paw inflammation [24]. On the other hand, these results contrast with a study showing increased nociceptive behavior in 18-month-old rats in the formalin test [25]. The discrepancy in the formalin test might be explained by age and species differences. Old mice in our experiments were 12 months of age in comparison to rats at 18 and 22 months in the other study [25].

A number of glucocorticoid targets are involved in the metabolism and immune response, including members of the NF-κB activation cascade [12,13,26]. One such target of glucocorticoids is the NF-κB-inhibitor protein alpha (IκBα) [15,16], which had higher expression in the spinal cord of formalin-treated old mice in comparison to young mice, indicating inhibition of NF-κB activation. The inducible isoform of nitric oxide synthase (iNOS) constitutes another glucocorticoid target, which is negatively regulated by cortisol and involved in immune reactions [27,28]. In our experiments, iNOS expression was significantly reduced in the spinal cord of old mice 45 min after formalin injection, which is in accordance with the simultaneously increased cortisol levels. However, since iNOS expression is also regulated by NF-κB [29,30], it is not clear if cortisol inhibits iNOS directly through activation of transcriptional glucocorticoid receptors or indirectly by inhibition of NF-κB activation.

Taken together, our results demonstrate an age-related decrease in inflammatory nociception, which is at least partially due to increased formalin-induced cortisol quantities in one-year-old mice. The increased glucocorticoid levels might inhibit activation of proinflammatory signaling pathways including NF-κB and the inducible NO synthase. However, it has to be kept in mind that age-related changes are complex and that a number of further mechanisms might also participate in the observed reduction of the inflammatory noxious response. Nevertheless, it is clear that a single study with a restricted number of mice only allows us to make limited conclusions about the physiological responses to age. Furthermore, it is also apparent that data from animal studies cannot be directly translated into the clinical context in humans.

## 4. Experimental Section

### 4.1. Animals

C57BL/6 mice, aged 6–8 weeks or 12 months, were used in all experiments. In the experiments concerning signal transduction we used male mice only. In the zymosan test we also used male mice exclusively; in the formalin test both male and female mice were used in the ratio 1:1 due to a limited number of old male mice bred in our own animal facility. However, separated analyses of both genders revealed no differences in the nociceptive response. Animals were subjected to behavioral testing and sacrificed at the indicated time points. Spinal cords were dissected out, immediately frozen in liquid nitrogen, and then stored at −80 °C until further preparation. In order to exclude an influence of cortisol’s circadian rhythm, all preparations for cortisol analyses were performed at the same time of day. In all experiments, the ethical guidelines for investigations in conscious animals were obeyed and the procedures were approved by the local Ethics Committee for Animal Research (Regierungspräsidium Darmstadt, F95/53). All efforts were made to minimize animal suffering and to reduce the number of animals used.

### 4.2. Behavioral Testing

#### 4.2.1. Motor Coordination (Hanging Wire Test) (Adapted to [31])

Motor coordination was assessed by placing mice on a grid that was turned upside down and held in this inverted position for 90 s (latency period). If mice were able to remain on four limbs in the hanging position for at least 90 s, their motor coordination was rated as intact.

#### 4.2.2. Mechanical Sensitivity (Dynamic Plantar Test)

Paw withdrawal latency to mechanical stimulation was assessed with an automated testing device consisting of a steel rod that is pushed against the plantar surface of the paw with increasing force until the paw is withdrawn (Dynamic Plantar Aesthesiometer, Ugo Basile, Varese, Italy) [32]. The maximum force was set at 5 g to prevent tissue damage and the ramp speed was 0.5 g/s. Mice were placed in test cages with a metal grid bottom. They were kept in the test cages for 1 h to allow accommodation. The paw withdrawal latency (PWL) was obtained as the mean of six consecutive assessments at each time point (at least 10 s between repeated measurements of the same paw).

#### 4.2.3. Thermal Sensitivity (Hargreaves Test) [33]

Paw withdrawal latency to thermal stimulation was assessed with an automated testing device consisting of a light ray that is directed against the plantar surface of a mouse’s hind paw after placing the mouse on a 32 °C warm transparent surface area (Plantar Aesthesiometer, Ugo Basile, Varese, Italy). Mice were kept in the test cages for 1 h to allow accommodation. Before the actual measurement takes place with a measuring light ray (intensity 13), the stand-by light ray (intensity 5) is positioned under the mouse’s hind paw manually and the measuring light ray is switched on. The time until the mouse withdraws the paw due to the thermal stimulus is analyzed (PWL). If the mouse shows no reaction within 20 s (cut-off time), the light ray is removed. The paw withdrawal latency was obtained as the mean of six consecutive assessments at each time point (at least 10 s between repeated measurements of the same paw).

#### 4.2.4. Hot-Plate Test [34]

Animals were placed in a Plexiglas cylinder on a heated plate maintained at 52 ± 0.2 °C (Ugo Basile), and the latency to jump or to shake/vibrate a hind paw was recorded. Each animal was tested only once, since repeated testing in this assay can lead to latency changes [35]. The cut-off time was 30 s.

#### 4.2.5. Acetone Test

To assess cold allodynia, a drop of acetone was applied with the help of a 1-mL syringe onto the plantar side of one hind paw. The time the mice spent lifting, shaking, or licking the acetone-treated paw was recorded with a stop watch during an observation period of 2 min starting immediately after acetone application [32].

#### 4.2.6. Formalin Test

The formalin test was performed as described [36]. Mice were placed in a Plexiglas cage and were allowed to habituate for at least 30 min. Twenty microliters of a 5% formaldehyde solution (formalin) were injected subcutaneously into the dorsal surface of the left hind paw. The time spent licking the formalin-injected paw was recorded at 5-min intervals up to 45 min, starting immediately after formalin injection.

#### 4.2.7. Zymosan-Induced Paw Inflammation, Mechanical Hypersensitivity

Hind paw inflammation was induced by subcutaneous injection of 20 µL of a 10 mg/mL zymosan A (Sigma-Aldrich, Munich, Germany) suspension in phosphate-buffered saline (0.1 M, pH 7.4) into the mid plantar region of the left hind paw [37]. Baseline mechanical sensitivity was determined before zymosan injection as described above (Dynamic Plantar Test). Mechanical hypersensitivity was analyzed as the mean of four consecutive assessments at 10 s intervals starting 1 h after zymosan A injection and then hourly up to 8 h.

### 4.3. Assessment of Cortisol Levels

The cortisol level in the spinal cord was investigated by a commercially available cortisol ELISA Kit (Antikörper-Online, Aachen, Germany, ABIN367750, Intra-assay: CV% less than 8% Inter-assay: CV% less than 10%) according to the manufacturer’s recommendations. The spinal cord of mice was dissected out with and without prior formalin treatment for 45 min. Tissues were homogenized by a mixer mill in phosphate-buffered saline and then centrifuged at 16,800× *g* for 20 min.

### 4.4. Reverse Transcription-Polymerase Chain Reaction (RT-PCR)

Total RNA was extracted from the indicated tissues as described previously [38]. Two hundred nanograms of total RNA were used for the reverse transcription, which was performed with the Thermo Scientific Verso cDNA system (Thermo Fisher Scientific GmbH, Darmstadt, Germany). Twenty nanograms of RNA equivalent were subjected to quantitative real-time PCR (qRT-PCR) in an Applied Biosystems Sequence Detection System AB7500 using SYBR Select Master Mix (Life Technologies, Darmstadt, Germany) with SYBR green fluorescence staining. Expression of PCR products were determined and normalized to glycerin aldehyde phosphate dehydrogenase (GAPDH) mRNA, which was detected in the same way. The following gene-specific primers for iNOS were used:

FW5′-CCAAGCCCTCACCTACTTCC-3′RV5′-CTCTGAGGGCTGACACAAGG-3′

iNOS and GAPDH primer were designed in our lab. TRPV1 and TRPA1 were determined by the use of pre-developed Taqman Assays (Life Technologies, Darmstadt, Germany). The primer kits are validated by the manufacturer. In addition, we have performed control experiments for all of the primers used and found only one band in the gels at the expected size, whereas the water controls showed no specific signal. The primers were used at a concentration of 1 pmol/µL; the Ct values were in the range of 31 for iNOS, 19 for GAPDH, 32 for TRPA1, and 25 for TRPV1. The cycle number at which the fluorescence signals cross a defined threshold (*C*_t_-value) is proportional to the number of RNA copies present at the start of the PCR. The threshold cycle number for the specific mRNA was standardized by subtracting the *C*_t_-value of GAPDH from the *C*_t_-value of the specific PCR product of the same sample. Relative mRNA quantities were determined by standard 2^(−∆∆*C*t)^ calculation and expressed as fold-change of reference samples (young mice).

### 4.5. Analysis of Nitrite/Nitrate

The release of NO was assessed by measuring concentrations of nitrite and nitrate using the Griess method [39]. Fifty microliters of spinal cord lysate were diluted with 150 µL H_2_O and mixed with 50 µL of 0.4% sulphanilamide in 1 N hydrochloric acid and 50 µL of 0.6% naphtylethylendiamine dihydrochloride in water. The absorbance of the mixture was measured photometrically at 540 nm. The reliable limit of quantification was 1 µM and the mean percentage deviation over the calibration range of 1–50 µM was less than 15%.

### 4.6. Western Blot Analysis

For Western blot analysis, tissues were homogenized in PhosphoSafe Extraction Buffer (Merck, Darmstadt, Germany) with protease inhibitor (1 mM Pefabloc SC, Alexis Biochemicals, Lausen, Switzerland). To remove cellular debris, extracts were ultracentrifuged at 16,800× *g* for 45 min at 4 °C. The supernatants were stored at −80 °C.

Protein lysates (30 µg) were separated electrophoretically by 10% SDS-PAGE and then transferred onto nitrocellulose membranes using a Trans-Blot Turbo Transfer System (Bio-Rad Laboratories GmbH, Munich, Germany). To confirm equal loading, all blots were stained with Ponceau red solution. Membranes were blocked for 60 min at room temperature in Odyssey blocking reagent (Licor, Bad Homburg, Germany) diluted 1:1 in 0.1 M PBS, pH 7.4. Then the blots were incubated overnight at 4 °C with primary antibody against IκBα (1:250, Cell Signaling Technology, Heidelberg, Germany) in blocking buffer. After washing three times in PBS with 0.1% Tween 20, the blots were incubated for 60 min with an IRDye 700-conjugated secondary antibody (Licor, Bad Homburg, Germany, 1:5000 in blocking buffer). After rinsing in 0.1% Tween 20 in PBS, protein–antibody complexes were detected with the Odyssey Infrared Imaging System (Licor, Bad Homburg, Germany). Heat shock protein 90 (Hsp90) (Sigma-Aldrich, Deisenhofen, Germany) was used as the loading control. Densitometric analysis of the blots was performed with Quantity One Software (Bio-Rad, Munich, Germany).

### 4.7. Data Analysis

Paw withdrawal latencies in response to mechanical stimulation were expressed as the relative difference between right control paw and the zymosan-treated left hind paw. The area under the time curve (AUC) was calculated by employing the linear trapezoidal rule. Statistical evaluation was done with SPSS 17.0 for Windows. Data are presented as means ± SD. Data were either compared by univariate analysis of variance (ANOVA) with subsequent t-tests employing a Bonferroni α-correction for multiple comparisons (Cortisol-ELISA) or by Student’s t-test (acute nociception (Table 1), Formalin test (Sum of licking time), Zymosan (AUC), IkappaB Western blot, Nitrate assay, iNOS-, and TRPV1- and TRPA1-RT-PCR). For analysis of inflammatory hypersensitivity in the zymosan-induced paw inflammation model and the formalin test repeated measures, ANOVA was performed. For all tests, a probability value *p* < 0.05 was considered statistically significant.

## 5. Conclusions

In conclusion, increasing age might have a beneficial effect on some types of inflammatory pain mediated, at least partially through a shift in the HPA axis towards increased cortisol release. Although this appears to be positive, it has to be taken into account that an ongoing increased exposure to glucocorticoids is associated with a wide range of side-effects and potential glucocorticoid resistance [40], which might affect physiological systems or anti-inflammatory therapies in the elderly.
